# A retrospective comparison of [^18^F]FDG radiation dose following a transition from conventional to long axial field of view PET/CT

**DOI:** 10.1007/s13246-025-01588-0

**Published:** 2025-07-18

**Authors:** Wei-Ting Jacky Chen, William I. D. Rae, Peter L. Kench, Kathy P. Willowson, Dale L. Bailey, Elizabeth A. Bailey, Heidi Fearnside, Eleanor Kelliher, Steven R. Meikle

**Affiliations:** 1 Discipline of Medical Imaging Science, Faculty of Medicine and Health, University of Sydney, Sydney, NSW Australia; 2 Medical Imaging Department, Prince of Wales Hospital, Sydney, NSW Australia; 3 Department of Nuclear Medicine, Royal North Shore Hospital, Sydney, NSW Australia; 4 Brain and Mind Centre, University of Sydney, Sydney, NSW Australia; 5 Sydney Imaging Core Research Facility, University of Sydney, Sydney, NSW Australia

**Keywords:** Positron emission tomography/computed tomography (PET/CT), Long axial field-of-view, Effective dose, ^18^F-FDG PET

## Abstract

Long axial field of view (LAFOV) PET/CT scanners (> 1 m axial FOV) provide an order of magnitude higher system sensitivity compared with conventional scanners. This creates opportunities for significant radiation dose reductions for patients, without loss of diagnostic image quality or increased scan time. This study aimed to investigate changes in radiation dose received by patients undergoing whole-body [^18^F]FDG PET/CT studies at a metropolitan hospital following the transition from the Siemens Biograph mCT (21.8 cm axial FOV) to the Siemens Biograph Vision Quadra LAFOV PET/CT (106 cm axial FOV). For the mCT and Quadra, 484 and 554 patient studies were reviewed, respectively. The radiation dose from the PET component was derived from the recorded FDG dose, calculated based on ICRP recommendations, and scaled to patient weight. The CT dose was derived from the dose-length product. The median effective dose from the PET component for the mCT and Quadra was 6.2 (IQR 5.5–6.9) and 2.9 (IQR 2.8–3.6) mSv, respectively, and 5.7 (IQR 5.1–6.5) and 2.8 (IQR 2.4–3.4) mSv, respectively, when scaled to patient weight. The median effective dose from the CT component for the mCT and Quadra was 7.7 (IQR 6.2–9.4) and 7.6 (IQR 5.9–9.4) mSv, respectively. The total median effective dose combining PET and CT components for the mCT and Quadra was 13.9 (IQR 12.4–15.7) and 10.5 (IQR 9.4–12.3) mSv, respectively, and 13.5 (IQR 12.4–15.0) and 10.3 (IQR 9.3–11.9) mSv, respectively, when scaled to patient weight. While the effective dose from PET was approximately halved due to reduced injected activity, the CT effective dose remained relatively unchanged and is now the dominant source of radiation dose to the patient for LAFOV PET/CT.

## Introduction

Long axial field of view (LAFOV) PET/CT scanners have been recently introduced which feature an axial field of view greater than 1 m for the PET component, resulting in approximately an order of magnitude higher PET system sensitivity compared to conventional PET/CT scanners [1]. Since PET and CT are both relatively high dose imaging modalities [2], the increased system sensitivity of LAFOV PET/CT provides opportunities to considerably reduce the radiation dose to patients during PET/CT examinations without loss of image quality or increase in imaging time. This may become particularly important as the range of clinical applications of LAFOV PET/CT expands beyond oncology, which is currently the dominant application of PET/CT in clinical practice. For example, studies have shown the potential for LAFOV PET/CT in the diagnosis and monitoring of autoimmune arthritides [3, 4], Parkinson’s Disease [5, 6], and infectious diseases such as COVID-19 [7, 8]. Studies using LAFOV PET/CT have also demonstrated its potential for cardiac [9], and whole-body perfusion studies [10].

While expanding the range of potential clinical applications is a primary motivation for the introduction of LAFOV PET/CT technology [11], it is also important to evaluate and minimise the radiation dose to patients, consistent with the principle of keeping the dose As Low As Reasonably Achievable (ALARA) [12]. This requires reducing radiation exposure from both the PET and CT components of the scan. For the PET component, this involves reducing the activity of the radiopharmaceutical administered to the patient. For the CT component, it involves altering the exposure factors to limit the radiation dose to the patient. While the system sensitivity of the PET component of LAFOV systems has increased, thus allowing a reduction in radiation dose without loss of image quality, the CT component has also seen improvements based on advances in CT technology and reconstruction algorithms, which may lead to further reductions in dose to the patient [13].

The purpose of this study was to retrospectively compare the radiation doses received by patients undergoing PET/CT scans on a LAFOV scanner with those received on a conventional PET/CT scanner. The study was conducted at the Royal North Shore Hospital where the Department of Nuclear Medicine recently made the transition to LAFOV technology in a collaborative partnership with the University of Sydney and the National Imaging Facility. It extends our previous study reporting radiation doses associated with low dose FDG protocols in whole-body PET/CT [14].

## Methods

An ethics waiver was obtained from the Deputy Director of Medical Services of Royal North Shore Hospital based on an assessment by Royal North Shore Hospital Medical Services classifying this as a quality improvement project.

### Conventional PET/CT studies

The conventional PET/CT dataset was derived from a study investigating the radiation dose received by patients during low-dose [^18^F]FDG protocols for whole-body PET/CT [14]. The scanner used for that study was the Biograph mCT S64 PET/CT scanner (Siemens Healthineers, Erlangen, Germany) (referred to as ‘mCT’). This scanner had four rings of LSO block detectors (13 crystals per block axially, each crystal 4 × 4 × 20 mm), providing a 21.8 cm axial field of view, and a bore diameter of 78 cm. The NEMA sensitivity, following the NU 2-2007 guidelines, is reported to be 9.7 kcps/MBq at the centre of the FOV [15]. This scanner included a Siemens SOMATOM Definition AS (referred to as ‘Siemens Definition AS’) 64-slice CT scanner with a rotation time of 0.33 s.

A total of 484 standard whole-body [^18^F]FDG PET/CT scans (273 adult males, 210 adult females, and 1 unknown gender) performed between October 2010 and August 2011 were included in the study. The standard dose for [^18^F]FDG was 250 MBq, increasing to 300 MBq for patients weighing more than 90 kg. The acquired axial scan range was from the vertex to mid-thigh, requiring 6–8 bed positions (87–113 cm) depending on the patient’s height. The standard acquisition time was 150 s per bed position, resulting in acquisition times ranging from 15 to 20 min. Images were reconstructed using an Ordered Subset Expectation Maximisation with Time of Flight and Point Spread Function Modelling (OSEM-TOF-PSF) algorithm with 3 iterations and 21 subsets. A Gaussian filter with a Full Width at Half Maximum (FWHM) of 5 mm was used.

All CT scans conducted on the mCT used the Siemens ‘CARE Dose’ package for automatic exposure control to minimise the radiation dose to the patient. The CT scans were acquired using a 120 kVp tube voltage, 80 mA effective reference tube current, and a pitch of 0.8. The slices were collimated with a 1.2 mm data channel thickness and reconstructed with a 3 mm slice thickness.

### LAFOV PET/CT studies

All the data for the LAFOV PET/CT component of the study were collected using the Biograph Vision Quadra PET/CT scanner (Siemens Healthineers, Erlangen, Germany) (referred to as ‘Quadra’). This PET scanner features 32 LSO detector rings (10 crystals per ring axially, each crystal 3.2 × 3.2 × 20 mm), providing an axial field of view of 106 cm, and a bore diameter of 78 cm. The average NEMA sensitivity of the system is 176 kcps/MBq [16]. The scanner includes a Siemens SOMATOM Definition Edge 128-slice CT (referred to as ‘Siemens DE’), which has a 78 cm aperture and a rotation speed of 0.28 s [17].

A total of 553 standard whole-body [^18^F]FDG PET/CT scans (319 adult males and 234 adult females), performed between late December 2023 and late February 2024, were included in this study. The standard activity injected was 150 MBq of [^18^F]FDG. The injected activity was not reduced further because handling very small volumes and having time-consuming dilutions would have potential increases in finger doses for the technologists. Furthermore, the nuclear medicine physicians preferred to take advantage of the sensitivity gain of the Quadra to produce higher quality clinical images while also considerably reducing scan times. For most patients, only one bed position was required because the extended axial FOV covers from the patient’s vertex to mid-thighs. However, for taller patients, a protocol using continuous bed motion (‘Flow-motion’) was employed resulting in an axial FOV longer than the standard 106 cm. The standard acquisition time was 5 min. Images were reconstructed using an OSEM-TOF-PSF algorithm with 4 iterations and 5 subsets. A Gaussian filter with a Full Width at Half Maximum (FWHM) of 2 mm was used. The Maximum Ring Difference (MRD) was 322 corresponding to an axial acceptance angle of 52°. This MRD, known as Ultra-high Sensitivity Mode, takes full advantage of the enhanced sensitivity of the Quadra.

Similar to the mCT, the CT scans conducted on the Quadra also used automatic exposure control (‘Care Dose’) to minimise the radiation dose received by the patient. The CT scans were acquired using a 120 kVp tube voltage, 80 mA effective reference tube current, and a pitch of 0.8. The slices were collimated with a 0.6 mm data channel thickness and reconstructed with a 3 mm slice thickness.

### Data analysis

To ensure comparisons between the two studies were meaningful, the data analysis methods used in the initial study were largely replicated. The data were analysed for the patients overall and for different weight ranges: less than 50 kg, 50–59 kg, 60–69 kg, 70–79 kg, 80–89 kg, 90–99 kg, and greater than 100 kg.

### PET dose

The effective dose from the PET component of the scan from the injected [^18^F]FDG activity (ED_PET_) was calculated using three different methods. The first method used the reported dose coefficients in ICRP publication 128 [18]. These factors were 0.019, 0.024, and 0.036 mSv/MBq for 70 kg adults, 57 kg adults, and 33 kg children, respectively. While there has been recent work in the literature arguing that the dose coefficient for the 70 kg adult is 0.017 mSv/MBq, this has not yet been published in an ICRP publication [19]. As such, the widely accepted 0.019 mSv/MBq value was used. Each patient was sorted into the reference weight that best matched their actual weight: for example, 70 kg adults for patients larger than 65 kg, 57 kg adults for patients that were 45–65 kg, and 33 kg children for patients less than 45 kg:

1 $$\begin{aligned}{ED}_{PET}&=Injected\,Activity\,\left(\hbox{MBq}\right)\\ &\quad\times Weight\,Factor\,(\hbox{mSv}/\hbox{MBq})\end{aligned}$$

To account for the actual weight of the patient, the ED_PET_ was scaled as follows:

2 $${ED}_{PET-WS}=\frac{{ED}_{PET}\times ICRP\,Model\,Weight\,(\hbox{kg})}{Patient\,Weight\,(\hbox{kg})}$$

However, this method of scaling to the patient weight is limited in its representation of the effective dose to the patient, particularly in larger patients where more of the radiation is absorbed by adipose tissue. An alternative method that scaled the effective dose to the blood volume of the patient was also used [14]:

3 $${ED}_{PET-BV}=\frac{{ED}_{PET}\times Indexed\,Blood\,Volume\,(\hbox{ml}/\hbox{kg})}{70\,(\hbox{ml/kg})}$$

where the patient blood volume is calculated based on the following [20]:

4 $$Indexed\,Blood\,Volume= \frac{70\,(\hbox{ml}/\hbox{kg})}{\sqrt{\frac{Body\,Mass\,Index\,(\hbox{kg}/\hbox{m}^{2})}{22\,(\hbox{kg}/\hbox{m}^{2})}}}$$

For the mCT and Quadra, the number of patients who had their height recorded, thus, allowing calculation of indexed blood volumes, were 387 and 134 patients, respectively. As such, there were fewer data available for the calculation of the PET effective dose scaled to blood volume for the Quadra cohort.

### CT dose

The effective dose from the CT component of the scan (ED_CT_) was calculated from the dose length product (DLP). Since all the patients in this study underwent standard whole-body scans, the effective dose per unit dose-length product conversion factor of 0.018 mSv/mGy.cm was used for conversion to CT effective dose [21]:

5 $${ED}_{CT}\left(\hbox{mSv}\right)=DLP(\hbox{mGy}\cdot\hbox{cm})\times 0.018(\hbox{mSv}/\hbox{mGy}\cdot\hbox{cm})$$

In addition to the above method, an alternative approach to determine the median effective dose from the CT component of the scan for all patients and each weight category was employed using CT-Expo (version 2.8). The settings used in CT-Expo were as follows (Table 1).

**Table 1:** The settings used in CT-Expo for the two CT scanners

Scanner model	Tube voltage (kVp)	Scan range (cm)	Total collimation (mm)	Table feed (mm)	Reconstructed slice thickness (mm)
Siemens definition edge (Quadra)	120	− 14 to 94	76.8	61.44	3
Siemens definition AS (mCT)	120	− 6 to 94	19.2	15.36	3
Siemens definition AS (mCT)	120	− 16 to 94	19.2	15.36	3

Since the scan range of the Siemens Definition AS was dependent on the number of bed positions of the PET component of the scan, two of the most common scan ranges were used

The scan range is based on the model in CT-Expo and covers the vertex (94 cm) to varying areas around the mid-thigh (− 16, − 14, − 6 cm). Since the scan range for the Siemens Definition AS was dependent on the patient height, the two most common scan ranges were used in the analysis. The table feed was the product of the total collimation and the pitch factor (0.8). Since the CT scanners used are all multi-slice scanners, the total collimation is calculated as the product of the slice collimation and the number of slices acquired simultaneously. As both CT scanners do not record the average mAs used, the median CTDI_vol_ for all patients and each weight category was used to estimate the corresponding mAs for each CTDI_vol_, following the instructions in the CT-Expo manual.

Since the gender of the patients for the mCT could not be determined from the dataset, the medians were obtained and processed in CT-Expo with the assumption that all patients were male for both cohorts (see Discussion for explanation). To check the potential error associated with this assumption on the median effective dose, CT-Expo was then configured for a female population. This involved a 5 cm caudal transposition of the scan range to maintain the same positioning as the male model. The effective dose to the patient was recorded using the tissue weighting factors from ICRP publication 103.

### Total effective dose

The total effective dose was calculated by adding the effective dose from the CT component to the PET component of the scan. This was done for all three different methods used for calculating the PET dose:

6 $${ED}_{Total}={ED}_{CT}+{ED}_{PET}$$

where ED_Total_ is substituted for ED_TOTAL-ICRP_ when using the dose coefficients in the ICRP publication, ED_TOTAL-WS_ when scaled by patient weight, and ED_TOTAL-BV_ when scaled using patient blood volume.

The patient weight, injected [^18^F]FDG activity, CT effective dose, PET effective dose, and the total dose were compared for all patients and for each weight category. The median was used for the comparison to minimise the impact of outliers on the results, particularly for larger and smaller patients and for instances where it is possible that the scanning parameters were affected by delays. Since the variances of the various parameters between the two samples were mainly unequal, the Mann–Whitney U test was used. The statistical tests were performed in R-studio version 4.3.2.

## Results

Table 2 shows a summary of the results comparing the medians of the mCT and Quadra cohorts for patient weight, injected activity, and effective doses for CT, PET, and PET/CT combined. A statistically significant difference was found between the weight of the two cohorts. However, it was less than 5% of the median patient body weight of either cohort. The injected activity was significantly lower for the Quadra cohorts compared with the mCT cohorts by 50.7%. This corresponds to the significant difference found between the PET effective dose of the mCT and Quadra cohorts for all methods of dose calculation, with the percentage difference ranging from a 50.9% decrease in the effective dose received for the Quadra cohort when scaling by patient blood volume to a 53.7% decrease in the effective dose received when using the ICRP model.

**Table 2:** Summary of the comparison between mCT and the Quadra for relevant dose parameters of all the patients

	mCT median (n = 484)	Quadra median (n = 553)	Percentage difference (%)	*p*-value
Weight (kg)	73	70	− 4.1	< 0.05*
Injected activity (MBq)	303	150	− 50.7	< 0.05*
ED_CT_ (mSv)	7.7	7.6	− 1.9	0.33
ED_PET_ (mSv)	6.2	2.9	− 53.7	< 0.05*
ED_PET-WS_ (mSv)	5.7	2.8	− 51.4	< 0.05*
ED_PET-BV_ (mSv)	5.6	2.7	− 50.9	< 0.05*
ED_TOTAL-ICRP_ (mSv)	13.9	10.5	− 24.8	< 0.05*
ED_TOTAL-WS_ (mSv)	13.5	10.3	− 23.6	< 0.05*
ED_TOTAL-BV_ (mSv)	13.5	10.5	− 21.9	< 0.05*

The number of patients’ ED_PET-BV_ and ED_TOTAL-BV_ recorded for the mCT and Quadra were 387 and 134, respectively

Table 3 shows the median weight of each weight category. No significant difference was detected when comparing the distribution of the mCT and Quadra cohorts for each weight category. The percentage of patients in the < 50 kg and 50–59 kg weight categories for the mCT and Quadra cohorts was 18.8% and 25.0% of the patients, respectively. Meanwhile, the percentage of patients in the 90–99 kg weight category for the mCT and Quadra cohorts was 13.0% and 7.8% respectively.

**Table 3:** Summary of the comparison between mCT and the Quadra for the patient weight of each weight category with the number of patients in each cohort listed

Weight category	mCT median weight (kg)	Quadra median weight (kg)	Percentage difference (%)	*p*-value
< 50 kg	45.9 (n = 16)	46 (n = 39)	0.2	0.81
50–59 kg	56 (n = 75)	56 (n = 99)	0	0.62
60–69 kg	65 (n = 110)	64.8 (n = 122)	− 0.4	0.29
70–79 kg	74 (n = 107)	74 (n = 126)	0	0.94
80–89 kg	83.5 (n = 78)	84 (n = 84)	0.6	0.28
90–99 kg	93 (n = 63)	93 (n = 43)	0	0.32
> 100 kg	108 (n = 35)	111.5 (n = 40)	3.2	0.18

### PET dose

**Fig. 1:**
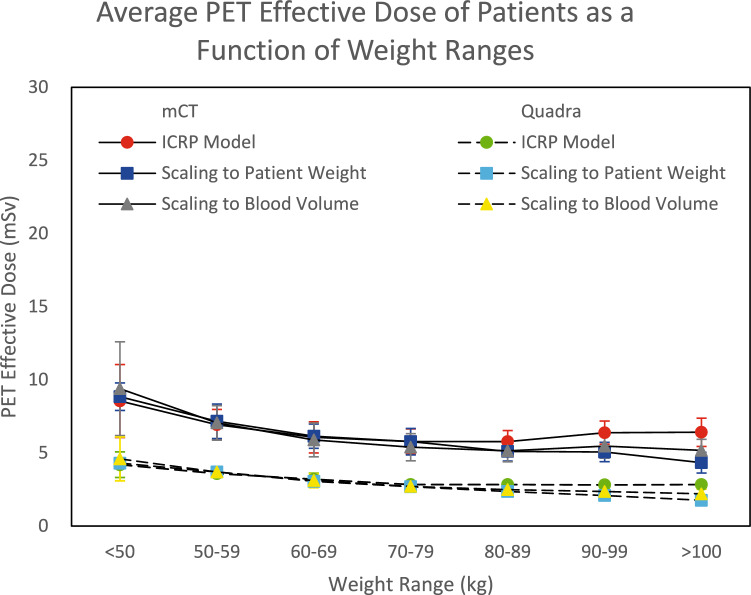
The average standard [^18^F]FDG PET effective dose received by mCT and Quadra patients as a function of weight range. The standard deviations are displayed as error bars

Figure 1 shows the total effective dose from the PET component of the scan as a function of weight range. The effective dose from the PET component of the scan was significantly lower (*p* < 0.05) for the Quadra cohort than the mCT cohort for all the weight categories for each method. This ranged from 47.6% lower when scaling to patient blood volume for the 50–59 kg and 70–79 kg weight categories to 60.0% lower for the Quadra cohort when scaling by patient weight for the > 100 kg weight category.

### CT dose

**Fig. 2:**
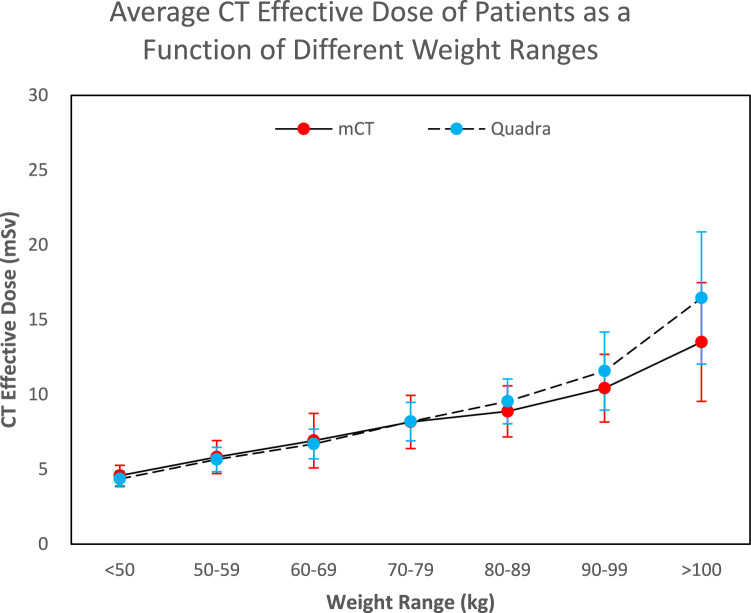
The average CT effective dose received by mCT and Quadra patients as a function of weight range. The standard deviations are displayed as error bars

Figure 2 shows the total effective dose from the CT component of the scan as a function of weight range. There was a statistically significant difference (*p* < 0.05) in the CT effective dose between the mCT and Quadra cohorts for weight categories 80–89 kg, 90–99 kg, and > 100 kg, where the dose was higher for the Quadra cohorts by 4.9%, 9.8% and 22.0% respectively. No significant difference in the CT effective dose between the two cohorts for the other weight categories was observed.

Table 4 compares the effective doses from the CT component of the scan for the mCT and Quadra cohorts. No significant difference was found overall between the mCT and Quadra cohorts for the effective dose received from CT. However, the median effective dose from CT was significantly higher for the Quadra cohorts for the weight categories 80–89 kg, 90–99 kg, and greater than 100 kg, with percentage increases of 4.9%, 9.8%, and 22%, respectively.

**Table 4:** The median effective dose of the CT component of the scan for the mCT and the Quadra cohorts separated into weight categories

Weight category	mCT ED_CT_ (mSv)	Quadra ED_CT_ (mSv)	Percentage difference (%)	*p*-value
< 50 kg	4.5 (n = 16)	4.4 (n = 39)	− 3.2	0.19
50–59 kg	5.6 (n = 75)	5.7 (n = 99)	0.9	0.76
60–69 kg	6.3 (n = 110)	6.6 (n = 122)	4.0	0.42
70–79 kg	7.7 (n = 107)	8.0 (n = 126)	4.7	0.15
80–89 kg	8.8 (n = 78)	9.2 (n = 84)	4.9	< 0.05*
90–99 kg	10.0 (n = 63)	10.9 (n = 43)	9.8	< 0.05*
> 100 kg	12.7 (n = 35)	15.5(n = 40)	22.0	< 0.05*

Table 5 represents the median CT dose obtained from CT-Expo. There was a slight decrease in the median CT effective dose after transitioning to the Quadra with the Siemens DE CT for the patient weight categories 60–69 kg, 50–59 kg, less than 50 kg, and all patients. there was a slight increase in the median CT effective dose after transitioning to the Quadra for the patient weight categories 80–89 kg and 90–99 kg. There was a larger increase in the median CT effective dose for the greater than 100 kg weight category after transitioning to the Quadra. No difference was observed in the median CT effective dose received by patients in the 70–79 kg category.

**Table 5:** Summary of results of the estimated dose using CT-Expo for the mCT over two different scan ranges, and the Quadra. The results are also separated into the different weight categories

Weight category (kg)	Siemens AS (mCT) median dose (100 cm) (mSv)	Siemens AS (mCT) median dose (110 cm) (mSv)	Siemens DE (Quadra) median dose (108 cm) (mSv)	% Difference between Siemens AS (100 cm scan range) and DE	% Difference between Siemens AS (110 cm scan range) and DE
< 50	3.1	3.1	2.7	− 15	− 15
50–59	3.8	3.8	3.5	− 9	− 9
60–69	4.2	4.2	4.1	− 2	− 2
70–79	4.9	4.9	4.9	0	0
80–89	5.5	5.5	5.7	4	4
90–99	6.2	6.2	6.6	6	6
> 100	7.2	7.3	9.3	23	22
ALL	4.9	4.9	4.7	− 4	− 4

Table 6 compares the average mAs and CTDI_vol_ from the median patient of each category derived from the CT-Expo analysis for the mCT and Quadra. Overall, the transition from the mCT to the Quadra resulted in an average mAs increase 15.8% and a CTDI_vol_ decrease of 0.8%. For the mAs and CTDI_vol_, a considerable 37.3% and 25.1% increase respectively, was observed after transitioning to the Quadra for the > 100 kg weight category.

**Table 6:** Comparison between the CT systems of the mCT and Quadra regarding the average mAs and CTDI_vol_ of the median patient in each weight category

Weight category (kg)	Siemens AS (mCT) average mAs	Siemens DE (Quadra) average mAs	Siemens AS (mCT) CTDI_vol_	Siemens DE (Quadra) CTDI_vol_	mAs % difference between Siemens AS and Siemens DE	CTDI_vol_ % difference between Siemens AS and Siemens DE
< 50	26.1	28.1	2.5	2.2	7.1	− 11.2
50–59	32.0	36.6	3.1	2.9	12.6	− 4.8
60–69	35.1	42.4	3.3	3.4	17.2	1.0
70–79	41.3	51.7	3.9	4.1	20.1	4.4
80–89	46.3	59.6	4.4	4.7	22.3	6.7
90–99	52.1	69.0	5.0	5.5	24.5	9.5
> 100	61.0	97.3	5.8	7.7	37.3	25.1
ALL	41.1	48.8	4.0	3.9	15.8	− 0.8

Table 7 compares the overall median CT effective dose of the male and female models using CT-Expo. The estimated effective dose using the female model is greater than the male model. The potential error for the mCT and Quadra are 16.9% and 16.1% respectively.

**Table 7:** Comparison of the CT effective dose using the male and female model in CT-Expo for the mCT and Quadra

	Male (mSv)	Female (mSv)	Potential error (%)
Siemens AS (mCT) median dose (100 cm)	4.9	5.9	16.9
Siemens AS (mCT) median dose (110 cm)	4.9	5.9	16.9
Siemens DE (Quadra) median dose (108 cm)	4.7	5.6	16.1

For a fair comparison, both the male and female models used the same scan length and exposure factors for each CT system as defined in Table 1

The proportion of the total effective weight-scaled dose that CT contributes for the mCT and Quadra cohorts is presented in Table 8.

**Table 8:** The proportion of the total effective weight-scaled dose that is from the CT component of a PET/CT scan

Weight-range	mCT (%)	Quadra (%)
< 50 kg	33	75
50–59 kg	49	64
60–69 kg	56	79
70–79 kg	61	65
80–89 kg	50	84
90–99 kg	68	72
> 100 kg	57	90

### Total effective dose

**Fig. 3:**
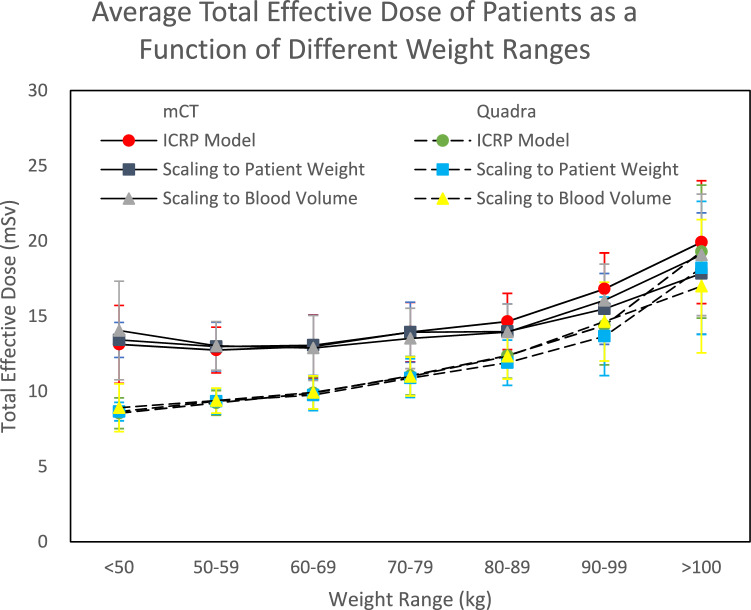
The average total effective dose (PET and CT component) received by mCT and Quadra standard [^18^F]FDG patients as a function of weight range, calculated using the ICRP model, scaling to patient weight, and scaling to blood volume. The standard deviation is displayed as error bars

Figure 3 shows the total effective dose from the PET and CT components of the scan for both cohorts (mCT and Quadra) as a function of weight range, with the PET dose calculated using the ICRP model, weight scaled, and blood volume scaled.

A significant difference was observed between the mCT and Quadra cohorts for the total effective dose received from PET/CT for all weight categories except for patients weighing greater than 100 kg. The median total effective dose was lower in the Quadra cohort, with a percentage decrease ranging from 15.3% for patients weighing 90–99 kg to 27.7% for patients less than 50 kg, when the dose was scaled by patient weight.

## Discussion

This study compared the effective radiation dose received by patients during standard whole-body [^18^F]FDG PET/CT examinations with those received during LAFOV PET/CT in a facility that recently transitioned from the former to the latter technology. While there were significant dose reductions observed for the PET component of the scan, the CT component of the scan was relatively unchanged between the conventional and LAFOV PET/CT.

The demographics of the mCT and Quadra patient cohorts were broadly similar as they were taken from the same metropolitan hospital. However, since the scans on the two cohorts were performed at least thirteen years apart, some differences in the demographics of the cohorts were noted. For example, there was a significant difference in the median patient weight between the mCT and Quadra cohorts (Table 2). This is because there was a considerably greater proportion of Quadra than mCT patients in the < 50 kg and 50–59 kg weight categories (Table 3). Furthermore, there was a considerably smaller proportion of Quadra than mCT patients in the 90–99 kg weight category. However, Table 3 shows that there was no significant difference in the patient weights within each weight category. This suggests that while the demographics of each weight category was unchanged, a greater percentage of patients with a smaller body habitus was present in the Quadra cohort than the mCT cohort. Since the statistical analysis used was based on medians, the impact of the overall weight difference between the two cohorts on the findings were limited.

The reduction in the injected [^18^F]FDG activity from a median of 303 MBq for the mCT cohort to 149.5 MBq for the Quadra cohort resulted in a correspondingly proportionate reduction in the radiation dose to the patient. As a comparison, the National Diagnostic Reference Levels (NDRL) of standard [^18^F]FDG examinations internationally are 400 MBq (United Kingdom) [22], 592 MBq (United States) [23], and 270 MBq (Australia) [24]. The estimated adult effective doses using the ICRP models from these reported activities are 7.6, 11.2, and 5.13 mSv, respectively. The median PET effective doses using the mCT from 2010 to 2011 reported in Table 2 are around 6.2 mSv, 1 mSv higher than the current Australian NDRL. Meanwhile, the median PET effective doses using the Quadra are much lower than the current NDRL at 2.9 mSv. This dose reduction is expected because the injected [^18^F]FDG activity has been reduced by approximately half. While the effective dose estimates for larger patients are not as accurately represented by the ICRP model, the significant reductions of the median injected activity by more than 50% suggests an expected associated considerable dose reductions to larger patients.

For the CT component of the scan, there were some scan parameter differences due to the change in scanner from the Siemens AS series to the Siemens DE. The scan range of the mCT was adjusted based on the number of bed positions required (usually 6–8 bed positions). As a result of the overlapping bed positions required for the PET component of the scan, the most common CT scan ranges were approximately 100 cm and 110 cm. The scan range of the Quadra did not change since only one bed position was required unless the patient was particularly tall. Therefore, the whole 106 cm scan range was used for both CT and PET. However, it should be noted that for some neurological scans in our cohort, the patient is positioned with the head near the centre of the axial field of view of the Quadra to maximise sensitivity. In this scenario, the CT only exposes the patient from the top of the head to approximately mid abdomen rather than below the pelvis as it would for other scans. Also, although both systems use the ‘CARE Dose’ package for AEC, the CT models are different. As such, it is reasonable to assume the methods of dose optimisation have been improved in the interim. Various CT manufacturers have developed their own iterative reconstruction algorithms that are designed to reduce the artifact and noise in the image, such as SAFIRE for Siemens, AIDR 3D for Toshiba/Canon, Veo for GE, and IMR for Philips [25]. However, there is limited information on the execution of these algorithms, and they are difficult to compare between CT models of different manufacturers given that the implementation methods are different between manufacturers [26]. A PET/CT phantom study by Brady et al. investigated the potential for iterative reconstruction for the CT component of the scan and found that when compared to filtered back projection only, dose reductions between 62 and 86% were possible [27]. However, beam hardening streak artifacts caused by arm bones, metal prostheses and tubing were more pronounced with more implementations of the iterative reconstruction algorithm over filtered back projection. The increased noise and artifacts are consistent with a recent study using an artificial intelligence iterative reconstruction (AIIR) algorithm for the uExplorer, another LAFOV PET/CT scanner [28]. The image generated by AIIR (10 mAs) was more obscure compared to the standard dose CT (120 mAs) for the brain and abdomen because of the attenuation from the skull and upper limbs. Despite this, it was found that implementation of the AIIR algorithm for PET/CT can be done under special circumstances for dose reduction. Despite these changes, using the DLP obtained directly from the automatically generated dose reports of the PET/CT scanner, the CT effective dose was not significantly different between the mCT and Quadra for the patient weight categories up to 80 kg (Table 4). However, there was a significant increase in the CT effective dose when using the Quadra compared to the mCT when the patient weight was 80–89 kg, 90–99 kg, and greater than 100 kg respectively. Despite the significant differences observed, since the ICRP model is not as robust for larger patient weights, the error on this data is large (Fig. 2). As such, the medians of the CT dose were also compared using CT-Expo. The patient categories from 50 to 99 kg all demonstrated very slight changes in the CT effective dose after transitioning from the mCT to the Quadra. Notably, the data suggest a CT effective dose reduction for patients weighing 50–59 kg. While the differences for patients weighing less than 50 kg and greater than 100 kg are greater, it must be noted that these weight categories are prone to the effects of extreme outliers where patients weigh much less than 50 kg or much more than 100 kg, especially because of the smaller sample size compared to the other weight categories. The effect of these extreme outliers seemed to also result in a substantial increase in the mAs and CTDI_vol_ following the transition from the mCT to the Quadra for the > 100 kg weight category. Also, despite the increases in mAs and CTDI_vol_, the CT effective doses of the patients were mostly similar. As such, the differences in CT effective dose between the mCT and Quadra are limited. Also, since the data on the gender of each mCT patient was no longer available, the gender of all patients were assumed to be male when analysing the data using CT-Expo. This is because the recorded scan range for the standard whole-body [^18^F]FDG was consistent with the male model size. There were key differences between the effective doses of the male and female models because CT-Expo did not provide a tissue weighting factor for male breasts and there were considerable differences in the organ doses of the testes compared to the uterus and ovaries. Therefore, the potential error calculated in Table 7 is expected. Further consideration should be placed on the effective radiation dose of females in CT. Therefore, for a more accurate comparison of the median CT dose to the patient between the mCT and Quadra, it could be beneficial to obtain the dose received by each patient while accounting for the patient’s gender and individual scan range using third-party CT dosimetry software such as CT-Expo. Also, given the significant dose reductions when implementing iterative reconstruction technology described in the literature, further investigations to optimise the patient effective dose from the CT component of the scan by using these algorithms, such as SAFIRE for the Biograph Vision Quadra, would be beneficial.

The total median effective dose for all patients in the mCT and Quadra cohorts were 13.9 and 10.5 mSv, respectively, when calculated using the ICRP model, 13.5 and 10.3 mSv when scaling for weight, and 13.5 and 10.5 mSv when scaling for blood volume. As a result of the considerable reduction in the PET effective dose, but little change, if any, in the CT effective dose to the patient after transitioning from the mCT to the Quadra, it is evident that the CT component of the scan now constitutes a greater proportion of the radiation dose to the patient (Table 8). When comparing all patients irrespective of weight categories, the contribution of CT to the total effective dose for the mCT and Quadra cohorts is 56% and 73%, respectively. This is because, regardless of the patient’s weight, the Quadra can scan patients using an injected activity of 150 MBq without loss of image quality, while the mCT required a higher standard injected activity of 250 MBq to maintain diagnostic image quality and 300 MBq for patients greater than 90 kg. Additionally, there were clinical trials running on the mCT which required a higher injected activity, whereas no such clinical trials had started for the Quadra at the time of this study.

The PET effective dose estimates reported are similar to previous measurements in the literature. Kaushik et al. estimated the dose received by males from an injected [^18^F]FDG activity of 370 MBq to be 7.4 mSv using an effective dose coefficient of 0.020 MBq/mSv, while the effective dose from the CT component was 6.8 mSv, totalling 14.2 mSv [29]. Quinn et al. found higher levels of radiation dose received by the patient, with a mean PET effective dose of 9.0 ± 1.6 mSv from an injected activity of 450 ± 32 MBq, and a mean CT effective dose of 15.4 ± 5.0 mSv, resulting in a combined effective dose of 24.4 ± 4.3 mSv [30]. There are also studies in the literature using injected [^18^F]FDG activities similar to the activity currently used at our institution for the Quadra. Zaman et al. found that the median effective PET dose for patients injected with a median dose of 194 MBq was 3.69 mSv, combined with a median CT dose of 4.93 mSv, resulting in a combined effective dose of 8.85 mSv [31].

This study demonstrates that the radiation dose to the patient can be reduced either by further reducing the injected [^18^F]FDG activity or by reducing the effective dose from CT, which have barely changed between the two cohorts despite the update in CT systems. Further reductions in the injected [^18^F]FDG activity are possible due to the substantially higher system sensitivity of the Quadra. Huang et al. have proposed acquisition parameters suitable for LAFOV PET/CT where the injected activity can be as low as 0.98 MBq/kg, which when compared to the full 3.70 MBq/kg dose, did not significantly affect the maximum SUV of large or small lesions, and the mean SUV of various healthy organs and tissues [32]. When comparing LAFOV PET/CT with an injected [^18^F]FDG activity of 1.85 MBq/kg using 2-min of acquisition time to conventional PET/CT, it was found that the lesion detectability was similar while the liver SNR was significantly greater for the LAFOV PET/CT system [33]. However, dose reduction is different to dose optimisation so care must be taken when pursuing low dose protocols as it masks the potential use for higher quality and lower noise images of the LAFOV compared to the conventional PET/CT [34]. For reductions in the CT effective dose, various methods are available. Optimising the protocol by appropriately identifying whether the CT scan is necessary for diagnostic purposes, anatomical localisation or only attenuation correction is essential to minimise the effective dose received by patients [26]. When compared to diagnostic scans, those for anatomical localisation and attenuation correction can obtain CT dose diagnostic levels that are reduced by 50–80% and 10–100 times, respectively [35]. Further acquisition techniques are viable for reducing the patient effective dose for attenuation correction. Cheng et al. reported in a phantom study that the dose from CT could be reduced to 0.3 mSv for attenuation correction using 100 kVp, 10 mA, 0.5 s rotation time, and a helical pitch of 0.984 [36]. Other methods recently explored with the introduction of the LAFOV PET/CT include the use of a tin filter, which could reduce the dose to the patient by 60–87% compared with ultra-low-dose CT [37]. This is similar to a more recent study that also demonstrated the use of tin filters to achieve a 90% dose reduction for CT attenuation correction for patients with a BMI lower than 30 [38]. Another method uses deep learning trained on image pairs with and without attenuation correction, to enable CT-less attenuation correction of the PET data to be performed [39]. Alternative methods of dose reduction have been proposed whereby the CT scan and/or the resulting attenuation map is replaced by synthesized data. For example, the maximum likelihood activity and attenuation (MLAA) algorithm estimates both the PET image and its corresponding attenuation map directly from the emission data based on TOF-PET consistency conditions [40]. This method also reduces image artifacts due to patient motion and high-density implants. However, there are some challenges with MLAA, including longer reconstruction time, the need for strong priors to constrain the solution and accurate scanner calibration and scatter estimates [41]. More recently, AI-based attenuation map prediction using PET data has demonstrated the potential for creating synthesized data [42]. However, there are also some challenges with this approach. For example, AI-generated attenuation images based on PET data typically exhibit blurring around the lungs because of respiratory motion [43]. Alternatively, one can use the background radiation emitted by ^176^Lu, a constituent of the LSO detectors in the PET scanner, as a transmission source for attenuation correction, a method made more viable with the large amount of detector material in a LAFOV PET/CT scanner [44]. Considering that LAFOV PET/CT systems have only recently become commercially available, further CT dose reductions are expected. When combined with the dose reductions from the PET component, this would result in significant overall dose reductions for LAFOV PET/CT compared to conventional PET/CT.

## Limitations

This retrospective study was conducted at a single-site and focused primarily on the mCT and Quadra PET/CT scanners. PET protocols for LAFOV PET systems are highly variable, dependent on the local preferences of the clinicians and patients. Therefore, even facilities using the same PET/CT scanner could potentially have different injected activities, acquisition times and image quality requirements. Despite these differences, the results of this study demonstrated a significant reduction in the PET effective dose to the patient without neglecting key improvements in acquisition time and image quality. The findings on the effective dose from the CT component should generalise to other LAFOV PET/CT sites with the caveat that some clinicians may request the CT component of the scan to be used for diagnostic purposes, which would increase the effective dose to the patient. The generalisability of the CT component is also affected by the features available on the CT scanner. Certain manufacturers may offer reconstruction algorithms with superior noise reduction, such as AI-based methods, which enable further reductions in the effective dose to the patient. These issues are no different than those currently experienced with conventional PET/CT. However, the discussed CT-less dose reduction methods would greatly benefit from the system sensitivity improvements LAFOV has over conventional PET.

## Conclusion

Long axial FOV PET/CT offers clinicians the potential to significantly reduce the radiation dose to patients. In this study, the primary source of the dose reduction on the Quadra LAFOV scanner was the PET component of the scan. By halving the median injected [^18^F]FDG activity, the PET effective dose to patients was also approximately halved. The CT effective dose remained relatively unchanged and has now become a greater contributor to the overall radiation dose in LAFOV PET/CT examinations, but this is anticipated to change with upgrades to the image reconstruction algorithms and technology changes envisaged for the future.
